# The Relation of Noises to Health Again

**Published:** 1893-10

**Authors:** 


### THE RELATION OF NOISES TO HEALTH AGAIN.

In the issue of the Journal for July, we discoursed briefly upon the influence that noises in great cities have over the health of their inhabitants, intimating that we should probably return to the subject again in the near future. We have also alluded, in paragraphs at various times, to the nuisances of certain particular noises. Possibly the subject may be somewhat threadbare to many of our readers. Nevertheless, it is one of great importance, and we venture to recur to the subject for the reason that reform can only be brought about by continual hammering at the evils which exist.

The *Medical News*, of Philadelphia, in its issue of August 26th, has considered the subject in much detail, and with great completeness, in an editorial entitled Medicine and City Noises. This article has attracted great attention among the newspapers in several American cities, many of which have published lengthy editorials on the subject. The *New York World*, in its issue of August 27th, was the first to take up the cudgel, adopting the editorial of the *News* as its text, and amongst those later to discourse thereupon, is the *Buffalo Enquirer*, in its issue of September 14th.

We herewith reproduce, from the *Medical News*, its editorial entire, and ask from our readers its careful consideration. The matter is ably presented, and is withal so scientific, that we hope the constituted authorities may heed the suggestions given and take some action to correct the evils complained of.

### MEDICINE AND CITY NOISES.

Not long since a foolish gentleman, who preferred to live in New York or not live at all, committed suicide rather than to longer endure the ear-splitting noise of the bells of a neighboring church. In thousands of cases people are being made ill, are committing slow suicide, or are being painfully and slowly killed by useless city noises. Noise, then, becomes a question of health and of medical importance concerning which physicians should have a word to say and a duty to perform.

Scientifically, our rebellion against the noise-makers is founded upon the physiologic truth that rest is necessary to health, and that over-stimulation or persistent stimulation of any organ or of all organs in essentially pathogenic. Herbert Spencer secured for himself a sad sort of freedom from noise by a mechanical contrivance that held some kind of soft plugs or stoppers in the external auditory meatuses. If there were only some method whereby one could, at will, shut out unwelcome sound, as one can shut out the light from the eyes ; if some aurist could devise an artificial method *à la* Spencer, one that would not injure the ear, he would be a great benefactor to humanity. The evil done the few people that would thereby possibly be burned to death or otherwise injured would be small, as compared with that of the many deaths and much sickness due to noise.

Sociologically, the whole community has an unrecognized duty as regards noise that rests upon a physiologic and æsthetic basis. Delicacy and accuracy of response to a physiologic stimulus are the characteristic marks of perfection in an organism. Whatever prevents this is against the welfare of society and progress. In this brutal noise-making era, one of two things must follow the ceaseless bruising of the mind by noise. Either the auditory mechanism, and the nervous mechanism with which it is related—that is, the whole mind—must become blunted in sensitiveness, crushed and stupefied ; or it must react pathologically. People are, therefore, divisible into two classes : those whose nervous systems and minds are becoming mechanicalized, anesthetic, and brutalized, and those who, thus failing to kill sense and mentality, develop disease-reactions. The distinct agency of noise is to make us either savage or sickly. Civilization, of which noise-making is a decided component, is thus bearing in its bosom a self-poison, to its own undoing. We are losing all refinement and delicacy of the senses, and are reverting to the condition of the barbarian whose senses had to be pounded and whipped into reaction, or we are becoming neurotic, hysteric, and neurasthenic. Generally and progressively, "Society" is either a crowd of the mentally stupid or of the hyperesthetically morbid, and social amusement is becoming a game of battering and spurring jaded and blunted senses, or of ministering to sense-diseases.

In the narrowest sense, we are medically bound to reduce the amount of noise-making, not only because noise engenders disease, but also because it prevents the cure of disease, or aggravates disease—very often, indeed, is the immediate cause of death. In an American city like Philadelphia, there are something like 3,000 needless deaths and the equivalent of 6,000 years of needless illness each year. What proportion of this waste of life is due to noise, it is, of course, impossible to say, but certainly a considerable proportion is chargeable to it. The sick are in private houses scattered all through the city, or in hospitals that are often located in the most densely crowded portions. Every physician knows how necessary quietness is to the sick, and how often noise has been the last baneful influence that the weakened organism could not resist, and thus the controlling and distinctive cause of failure to cure.

The recklessness of production and the unnecessariness of modern city noises are disgustingly astonishing. The worst of it is that they are even kept up throughout the night. If the night, at least, were kept quiet, and the organism were thus given periods of repose, it would not be so impossible to preserve normality of sense-reaction and sanity of mental reaction. In Philadelphia one or a dozen drunken brawlers may make the night hideous with howl, and curse, and obscenity. Remonstrance with the policeman elicits a smile, with ill-concealed contempt for the remonstrant as a crank, and the avowal that he has no authority to interfere. In Philadelphia it is illegal for locomotive-engineers to blow whistles, and yet all night long, sleep, at least in Summer, is to healthy ears and minds impossible by reason of this ear-splitting curse. Street-car bells rung all the time (by horses or by wheels) are no protection to the public, and yet the public submits to the horrible nuisance. The laying of railway-tracks and the paving of streets at night are only necessary for the advantage and profit of mercenary corporations, and yet the authorities have no power, or do not assert it, to repress the evil. If our freedom-loving American submits to the dictation of his tyrant and master as to trolley-cars, his master puts down the new tracks at night, with fiendish noises, and will by-and-by run the cars with the still more fiendish and ceaseless noises.

From whatever aspect the subject be considered, it seems strange that people will submit to the indignities of the noise-makers. A thousand are outraged in order that one or a few may possibly be benefited. The shrieking of whistles and the ringing of bells to notify workmen to stop or to start work is an instance in point. Everybody has a watch or a clock at hand. Why, then, blow the whistles? Why, also, thunder or jangle bells to tell people that should be asleep what o'clock it is during the night? The ten per cent. of people who go to church must be warned by bells; but have the ninety per cent. no rights who do not need or heed; and what about the sick? The milkman arouses a whole neighborhood in delivering a quart of milk. The cartmen, the peddlers, the hawkers, the ragmen, etc., bawl and howl to be heard half a mile away if some other greater noise near by did not drown their voices. There are persons that think it strange that barking dogs and crowing roosters in a city should be objected to.

All noises may be divided into the necessary, the partially necessary, and the wholly superfluous. The makers of the last class of noises should be proceeded against in the interests of the public health by all the forces and with all the vigor at the command of physicians. And this by all odds is the largest and most injurious class of noises. Here is a work ready for the Associations for the Public Good. There is something particularly exasperating and baneful in the unnecessary noise in the very fact of its unnecessariness. Let all the loafing rowdies, howlers, hawkers, whistle-blowers, bell-ringers, and the rest be incontinently hushed, and especially if they carry on their diabolism at night.

Concerning the class of partly preventable noises of cities, the greater amount of them is connected with street traffic, and here arises the shameful need of good smooth pavements. As with the strawberry so it is with the asphalt pavement—doubtless a better one could have been or may be invented, but doubtless it never has been invented. It is incomprehensible that people should consent to endure the torment arising from the stone and boulder pavements, and seemingly designed, like African music, for creating the most intolerable clatter possible. In addition to this aspect of the question, there is another reason why, as physicians, we should do away with block and cobble-stone pavement; they are excellent culture-grounds for lodging filth and disease-germs. The asphalt pavement offers no such a nidus, and can be easily flushed and kept clean.

The degree and character of the civilization of a country are indicated by the amount of unnecessary noise it endures, and this is accurately gauged by the condition of the pavements of its cities.

In the city of Buffalo, the noise incident to pleasure and traffic is greater, we venture to assert, than that required for the same conduct of affairs in the city of London, and it is assuredly greater than in the city of Paris. We mean that the aggregate noise in Buffalo is greater than the aggregate in either of the cities mentioned, both of which are several times larger than Buffalo. So long as the question of noise is considered only as a mere discomfort, no one would feel disturbed enough over it to call the attention of the municipal authorities thereto, but when the noise nuisance becomes so serious as to menace health, it is high time that measures were adopted to at least reduce it to a minimum. Every superfluous noise, from the shouting, tooting, and whistling boy, up to the ringing of church bells and screeching of locomotives and other steam whistles, should be prohibited under severe penalty.

In a nation whose nerves are so highly strung as ours, there is sufficient unavoidable wear and tear in the every-day conduct of affairs to shorten human life without the addition of a large catalogue of unnecessary noise nuisances. We respectfully invite the attention of the health authorities of Buffalo to this important subject. Preventive medicine has an important function to perform in the field of noise nuisance.

